# Impacts of self-confidence cultivation combined with family collaborative nursing on the hope level, stigma and exercise tolerance in patients undergoing radical resection of pulmonary carcinoma

**DOI:** 10.3389/fsurg.2023.1095647

**Published:** 2023-05-24

**Authors:** Fei Ye, YouHong Wu

**Affiliations:** Department of Cardiothoracic Surgery, Lishui Central Hospital, Lishui, China

**Keywords:** self-confidence cultivation, family members’ collaborative nursing, radical operation for lung cancer, hope level, stigma, exercise tolerance

## Abstract

**Objective:**

To analyze the impacts of self-confidence cultivation combined with family collaborative nursing on the hope level, stigma and exercise tolerance in patients undergoing radical resection of pulmonary carcinoma.

**Methods:**

In this expeirment, 79 patients who underwent radical resection of pulmonary carcinoma in our hospital from January 2018 to December 2021, were selected as research objects, and they were divided into two groups according to the date of admission. The control group (*n* = 39) was given routine care, while the study group (*n* = 40) was given self-confidence cultivation combined with family collaborative nursing on the basis of the control group. The hope level, stigma, exercise tolerance, and cancer-related fatigue of the two groups were comparatively analyzed.

**Results:**

The scores of T, P, I dimensions in Herth Hope Inventory (HHI) as well as the total score in the two groups were higher after intervention than before intervention (all *P* < 0.05).The scores of T, P, I dimensions and total scores of the HHI in the study group were higher as compared with the control group (all *P* < 0.05). After intervention, the scores of each dimension of the Chinese version of the Lung Cancer Stigma Scale (CLCSS), the modified British Medical Research Council Dyspnea Scale (mMRC), and the scores of each dimension of the Cancer Fatigue Scale (CFS) were lower than before intervention (*P* < 0.05); the 6-min walk test (6 MWT) result was longer than before intervention (*P* < 0.05); the scores of each dimension of CLCSS scale, mMRC score, and each dimension of CFS scale in the study group were lower as compared with the control group (*P* < 0.05) (*P* < 0.05).

**Conclusion:**

Self-confidence cultivation combined with family collaborative nursing can promote the hope level of patients undergoing radical resection of pulmonary carcinoma, reduce stigma, enhance exercise endurance, and relieve cancer-related fatigue.

## 1. Introduction

With the increasing morbidity and mortality, lung cancer has posed a big threat to people's lives and health (1). With the development of the times, the diagnosis and treatment technology of lung cancer is changing with each passing day, and the number of early diagnosed cases of lung cancer is increasing. Radical resection of lung cancer refers to the complete removal of tumor and local lymph nodes in patients with lung cancer (2). It has been reported that the physical recovery of patients undergoing radical resection of pulmonary carcinoma depends on the operation effect and nursing effect to certain extent (3). Therefore, it is necessary to pay attention to perioperative nursing care of patients undergoing radical resection of pulmonary carcinoma. Generally, people are “afraid of cancer and eager for staying health”. Influenced by the saying that “cancer is a terminal disease”, it is easy for patients to have psychological barriers or lose courage to try to treat cancer. With the development of medicine, cancer is no longer an “incurable disease”. Lack of self-confidence and negative treatment is a major reason limiting cancer treatment and rehabilitation. Patients undergoing radical resection of pulmonary carcinoma generally lack self-confidence in rehabilitation. Self-confidence cultivation emphasizes comprehensive use of various means to effectively enhance patients' self-confidence in overcoming the disease (4). Family-based collaborative nursing is a nursing method developed on the basis of responsibility-based nursing, which emphasizes the participation of patients' families in the nursing process (5). In this experiment, patients undergoing radical operation of lung cancer were given self-confidence training combined with family-based collaborative nursing.

## 2. Patients and methods

### 2.1. Patients

This experiment was approved by the Ethics Committee of the Hospital (approval number: 2022389). Inclusion criteria: ① Diagnosis of lung cancer (6); ② Indications for radical operation of lung cancer; ③ Clear consciousness; ④ Expected survival time ≥6 months; ⑤ Informed consent. Exclusion criteria: ① Diseases of important organs (heart, liver and kidney); ② Coagulation disorder; ③ Complicated with other malignant tumors; ④ Cognitive impairment, or mental illness. In this experiment, 79 patients who underwent radical resection of pulmonary carcinoma in our hospital from January 2018 to December 2021 were selected as research objects, and they were divided into two groups according to the date of admission. There were no significant differences in general data (age, pathological type, etc.) between the control group (*n* = 39) and the study group before intervention (*n* = 40) (*P* > 0.05).

### 2.2. Methods

The control group was given routine nursing for 6 months. The contents of routine nursing include: ① monitoring vital signs; ②using the method of transferring pain, or giving analgesic drugs correctly and timely according to the doctor's advice; ③ Choosing high-protein, high-calorie and digestible foods according to patients' dietary preferences; (4) assigning specialized personnel responsible for daily ward cleaning, ventilation and disinfection and keeping appropriate temperature and humidity; ⑤introducing the knowledge of prevention and treatment of lung cancer to patients through various channels (oral education, broadcast education videos, etc.); ⑥ Carrying out lung rehabilitation training (such as lip contraction breathing, etc.).

On the basis of the control group, the study group was given self-confidence cultivation combined with family-based collaborative nursing for 6 months. (1) Self-confidence cultivation: ① Direct experience. Instruct patients to accurately master the skills of exercise and self-care, giving timely verbal praise to patients who perform well, and assist patients to jointly formulate phased exercise rehabilitation goals. ② Vicarious experience. Obtain experience in disease resistance *via* communicating with patients, and learning exercise skills at the same time; the department can also organize patients to watch missionary videos together and exchange experiences. ③ Oral support. In the process of communicating with patients, motivational words should be used more to help patients overcome problems. ④ Emotional guidance. Guide patients to express their true feelings, listen to patients' complaints, find the root of their psychological problems, give appropriate interventions considering patients' personality characteristics and cognitive level, and teach emotional adjustment methods, such as music therapy and mindfulness training. (2) Family-based collaborative nursing: ① Collaborative living guidance. Introduce the treatment and rehabilitation programs to the patients' families, instruct the patients' families to actively communicate with the patients and understand their physical conditions, and truthfully record any discomfort. Eat eggs, chicken, and legumes as daily meals, and reasonably increase qi-tonifying and blood-nourishing foods (such as red dates). ② Coordinated movement guidance. The patients' family members should supervise the implementation of patients' exercise plan, and record patients' daily exercise time and exercise intensity. ③Collaborative psychological guidance. families should talk more with the patients to give them emotional support, and share more positive things to increase their positive emotional experience. Regularly accompany patients to participate in patient-friend communication meetings, allow them to share and exchange experiences with each other. ④ Collaborative health education guidance. Every Saturday at 9: 00 a.m., patients and one of their families are invited to participate in health lectures organized by the department to popularize the knowledge of lung cancer prevention and treatment. At the same time, it is necessary to establish regular contact between patients' families and nurses, guide patients' families to participate in the daily care program, and strengthen family support.

### 2.3. Observation indicators

(1) Comparison of hope level. The evaluation of the Herth Hope Index (HHI) (7) before and after intervention was carried out. HHI consists of 3 dimensions and 12 items, including ① positive attitude towards reality and future (T); ② taking a positive attitude (P); ③ keeping close relationship with others (I). Each item is evaluated with a scale of “1 point to 4 points”. The higher the score of each dimension of the HHI scale, the higher the hope level.
(2) Comparison of stigma. The evaluation of Chinese version Lung Cancer Stigma Scale (CLCSS) (8) before and after intervention was carried out. CLCSS consists of 27 items in 4 dimensions, namely: ① Shame and disgrace (14 items); ② Social isolation (6 items); ③ Discrimination (3 items); ④ Smoking (4 items). All items are evaluated with a 4-point scale system from 1 point (strongly opposed) to 4 points (strongly agreed). The higher the score of each dimension of CLCSS, the stronger the stigma.
(3) Comparison of exercise endurance. All patients were evaluated by the Modified British Medical Research Council (mMRC) (9) before and after intervention, with scores ranging from 0 point (no dyspnea) to 4 points (dyspnea); In addition, the 6-minute walking test (6 min) was conducted.
(4) Comparison of cancer-related fatigue. Cancer-related fatigue scale (CFS)-based evaluation (10) was carried out before and after intervention. CFS consists of 15 items in 3 dimensions, namely: ① Cognitive fatigue (4 items); ② Physical fatigue (7 items); ③ Emotional fatigue (4 items). Each item was evaluated with a scale of “0 point (no fatigue) to 4 points (severe fatigue)”. The higher the score of each dimension of CFS, the more serious the fatigue.

### 2.4. Statistical analysis

SPSS 23.0 was used for data analysis. The measurement data are expressed as the mean ± standard deviation, with *t-*test carried out for difference comparison. The counting data (pathological type distribution and other data) are expressed by *n* (%), with chi-square test performed for difference comparison. The difference is considered statistically significant when *P* < 0.05.

## 3. Results

### 3.1. Comparison of hope level

The scores of T, P and I dimensions of HHI scale as well as the total scores of the two groups were higher after intervention than before intervention (*P* < 0.05). Moreover, the scores of T, P and I dimensions of HHI scale as well as the total score of the study group were higher than those of the control group (*P* < 0.05). Table 2.

**Table 1:** Comparison of general data.

Group	*n*	Gender (*n*)		Age $\left(\bar{x}\pm s\right)$, year	Pathological type (*n*)			TNM staging (*n*)	
		male	female		Adenocarcinoma	Squamous cell carcinoma	Adenosquamous carcinoma	Phase I	Phase II
study group	40	24 (60.00)	16 (40.00)	51.05 ± 6.54	13 (32.50)	23 (57.50)	4 (10.00)	27 (67.50)	13 (32.50)
control group	39	20 (51.28)	19 (48.72)	49.43 ± 7.72	14 (35.90)	19 (48.72)	6 (15.38)	24 (61.54)	15 (38.46)
*χ*^2^/*t*		*χ*^2 ^= 0.608		*t *= 1.007	*χ*^2 ^= 0.805			*χ*^2 ^= 0.307	
*P*		0.435		0.317	0.668			0.580	

**Table 2:** Comparison of hope levels ($\bar{x}\pm s$, score).

Group	*n*	T		P		I		Total score of HHI scale	
		Before intervention	After intervention	Before intervention	After intervention	Before intervention	After intervention	Before intervention	After intervention
Study group	40	7.44 ± 2.32	12.16 ± 1.29^*^	8.11 ± 2.08	13.04 ± 1.23^*^	8.58 ± 2.26	13.65 ± 1.14^*^	24.13 ± 6.66	38.85 ± 3.66^*^
Control group	39	7.92 ± 2.23	11.04 ± 1.52^*^	8.72 ± 2.39	12.16 ± 1.65^*^	8.04 ± 2.37	12.18 ± 1.76^*^	24.68 ± 6.99	35.38 ± 4.93^*^
*t*		0.937	3.534	1.211	2.692	1.037	4.417	0.358	3.558
*P*		0.352	0.001	0.230	0.009	0.303	0.000	0.721	0.001

Compared with the same group before intervention *P < 0.05.

### 3.2. Comparison of stigma

The scores of CLCSS scales of the two groups were lower after intervention than before intervention (*P* < 0.05). The score of CLCSS scale of the study group was lower than that of the control group (*P* < 0.05) Table 3.

**Table 3:** Comparison of stigma ($\bar{x}\pm s$, score).

Group	*n*	Shame and shame		Social isolation		Discriminate		Smoking	
		Before intervention	After intervention	Before intervention	After intervention	Before intervention	After intervention	Before intervention	After intervention
Study group	40	38.76 ± 5.32	30.35 ± 3.76^*^	18.88 ± 2.34	14.26 ± 1.42^*^	9.43 ± 1.22	7.32 ± 1.45^*^	11.54 ± 2.21	8.43 ± 1.42^*^
Control group	39	40.48 ± 4.95	33.27 ± 4.48^*^	19.42 ± 2.03	15.23 ± 1.86^*^	9.02 ± 1.35	8.13 ± 1.68^*^	11.02 ± 2.02	9.31 ± 1.64^*^
*t*		1.487	3.141	1.094	2.609	1.417	2.296	1.091	2.573
*P*		0.141	0.002	0.277	0.011	0.161	0.024	0.279	0.012

Compared with the same group before intervention *P < 0.05.

### 3.3. Comparison of exercise endurance

The mMRC scores of the two groups were lower after intervention than before intervention (*P* < 0.05), and 6 MWT was longer than that before intervention (*P* < 0.05). The mMRC score of the study group was lower than that of the control group (*P* < 0.05), and the 6 MWT of the study group was longer than that of the control group (*P* < 0.05).

### 3.4. Comparison of cancer-related fatigue

The scores of CFS scales in the two groups were lower after intervention than before intervention (*P* < 0.05), and the scores of CFS scales in the study group were lower than those in the control group (*P* < 0.05).

## 4. Discussion

Hope is a kind of “faith” that can help an individual overcome difficult situations. For cancer patients, hope can bring them the power to overcome their fear, face the disease squarely, and firm the belief in recovery (11). A person's hope level is related to its physical and mental health condition, and a high hope level can promote the living quality. Table 2 shows that self-confidence cultivation combined with family cooperative nursing can promote the hope level of patients undergoing radical resection of pulmonary carcinoma. This may be due to the fact that the cultivation of self-confidence can continuously enhance the survival desire of patients undergoing radical resection of pulmonary carcinoma, strengthen their belief in recovery, help them develop healthy living habits, and keep their mood stable during scientific anti-cancer activities. To some extent, such nursing scheme is beneficial for disease control and help promote the recovery of physiological functions. Family-based collaborative nursing can provide emotional support for patients undergoing radical resection of pulmonary carcinoma, and enhance their sense of belonging. As a result, patients' hope level will be improved with the enhancement of their physical and mental condition.

Foreign studies have showed that lung cancer patients may suffer from stigma. The stronger the stigma, the worse their living quality (12). Stigma refers to the shame experience generated when an individual is discriminated against by others because of his or her illness (13). Table 3 shows that self-confidence cultivation combined with family-based cooperative nursing can reduce the stigma of the patients undergoing radical resection of pulmonary carcinoma. The cultivation of self-confidence requires verbal support and emotional counseling, which helps patients relieve their emotions, and truly feel the concern from nurses. At the same time, direct experience and vicarious experience help patients to gradually build a correct behavior pattern and establish a correct cognition of lung cancer, so as to alleviate their stigma. In this experiment, patients undergoing radical resection of pulmonary carcinoma received strong emotional support from family-based cooperative nursing. They were not afraid of other people's comments and judgments, and more willing to open their hearts to others, so their stigma decreased with the enhancement of their confidence.

Attention should be paid to the recovery of exercise endurance of the patients undergoing radical resection of pulmonary carcinoma (14) Table 4 shows that self-confidence cultivation combined with family cooperative nursing can enhance the exercise endurance of the patients undergoing radical resection of pulmonary carcinoma. This may be due to the fact that the cultivation of self-confidence allows patients to cooperate with clinical diagnosis and nursing more actively. With the continuous improvement of self-confidence in rehabilitation, patients can adhere to long-term regular exercise according to the doctor's advice, which is of great significance to the improvement of exercise endurance. Family-based collaborative nursing is a comprehensive nursing mode integrating “nurses, patients and family members”. Under the continuous supervision of families, patients undergoing radical resection of pulmonary carcinoma are willing to take the initiative to participate in disease management, and carry out rehabilitation training scientifically and regularly, so that their exercise endurance can be improved.

**Table 4:** Comparison of exercise endurance ($\bar{x}\pm s$).

Group	*n*	mMRC (score)		6 MWT (m)	
		Before intervention	After intervention	Before intervention	After intervention
Study group	40	3.28 ± 0.34	2.22 ± 0.38^*^	222.78 ± 20.65	448.86 ± 14.74^*^
Control group	39	3.37 ± 0.27	2.53 ± 0.41^*^	230.34 ± 18.02	400.08 ± 16.39^*^
*t*		1.301	3.487	1.732	13.917
*P*		0.197	0.001	0.087	0.000

Compared with the same group before the intervention *P < 0.05.

Cancer-related fatigue is usually a subjective feeling caused by tumors and related treatments, which can reduce the living quality of patients (15). Table 5 shows that self-confidence cultivation combined with family cooperative nursing can alleviate cancer-related fatigue of the patients undergoing radical resection of pulmonary carcinoma. Cancer-related fatigue is related to the personality characteristics of. In this experiment, the cultivation of self-confidence can mobilize the inner positive psychological quality of the patients, allow them to actively cooperate with the diagnosis and nursing work, and continuously reduce the occurrence of cancer-related fatigue through self-efforts. Family-based collaborative nursing helps to improve the nursing ability of patients' families, mobilize patients' subjective initiative, strengthen their belief in health and overcoming diseases, and allow them to take diet and exercise regularly.

**Table 5:** Comparison of cancer-related fatigue($\bar{x}\pm s$, score).

Group	*n*	Cognitive fatigue		Physical fatigue		Emotional fatigue	
		Before intervention	After intervention	Before intervention	After intervention	Before intervention	After intervention
Study group	40	10.32 ± 2.79	6.34 ± 1.73^*^	20.48 ± 3.54	15.33 ± 2.57^*^	10.28 ± 2.84	5.11 ± 1.68^*^
Control group	39	10.04 ± 2.92	7.47 ± 2.26^*^	21.11 ± 3.42	17.45 ± 2.86^*^	9.95 ± 2.92	6.54 ± 2.33^*^
*t*		0.436	2.499	0.804	3.467	0.509	3.135
*P*		0.664	0.015	0.424	0.001	0.612	0.002

Compared with the same group before the intervention *P < 0.05.

In addition,Due to time and manpower constraints, only 79 patients undergoing radical resection of lung cancer in our hospital were selected as research objects in this study. The sample size was small and the source was limited, so the generalization of the conclusions obtained was limited to some extent. In addition, the intervention time in this study was limited to 6 months, which is relatively short, and the long-term intervention effect needs to be verified by further studies. Future studies suggest expanding the sample size and sample sources, exploring the intervention effect of self-confidence cultivation combined with family cooperative nursing in different regions and populations, extending the intervention time, conducting follow-up investigations, and evaluating the long-term intervention effect.

## 5. Conclusions

To sum up, self-confidence cultivation combined with family cooperative nursing can promote the hope level of the patients undergoing radical resection of pulmonary carcinoma, reduce their stigma, enhance their exercise endurance and relieve their cancer-related fatigue. It can improve the quality of life and prognosis of patients with radical resection of lung cancer, and prolong the survival time. In the future, further research with larger sample size and longer intervention time can be carried out to guide clinical work.

## Data Availability

The original contributions presented in the study are included in the article, further inquiries can be directed to the corresponding author.
